# From Preparedness to Recovery: Learning Lessons Of COVID-19 Outbreak from China

**DOI:** 10.24248/eahrj.v4i1.616

**Published:** 2020-06-26

**Authors:** Charles Nsanzabera, Leonard Ndayisenga, Jean Damascene Kabakambira, Felix Hagenimana

**Affiliations:** a Department of health science, School of Public Health, Jomo Kenyatta University of Agriculture and Technology, Kenya; b Project Medical Referent, Abdurafi Neglected Diseases and Outbreak response Project, Amhara regional state of Ethiopia, MSF Holland, Ethiopia Mission; c Department of Internal Medicine University Teaching Hospital of Kigali, Rwanda; d Department of Statistics and Actuarial Science, Jomo Kenyatta University of Agriculture and Technology, Kenya

## Abstract

At the end of December 2019, the Chinese public health authorities reported several cases of acute respiratory syndrome in Wuhan City, Hubei province, China. Chinese scientists soon identified a novel coronavirus as the main causative agent. The disease is now referred to as coronavirus disease 2019 (COVID-19), and the causative virus is called severe acute respiratory syndrome coronavirus 2 (SARS-CoV-2). The COVID-19 outbreak was declared a pandemic by the World Health Organization on March 12th, 2020. COVID-19 propagates quickly and threatens the population at large; around 20% of affected populations have presented severe forms of the diseases. In China approximately ~5% cases became critical patients in need of admission to intensive-care units. The need for intensive care has led to unprecedented overcrowding in hospitals, with catastrophic situations witnessed in Italy and other countries. The highest mortality rates have been witnessed amongst the elderly with several comorbidities. In this viewpoint we draw lessons from the implementation of population containment measures, vulnerable people protection and relevant public health pillars in China. We then discuss how these lessons can or cannot be applied to other settings.

## BACKGROUND

COVID-19 is a severe acute respiratory syndrome (SARS) with pandemic characteristics that was first reported in Hubei province, Wuhan City in central China in December 2019. ^1^ It is caused by novel coronavirus 2019 (nCoV-2019), one of the coronaviruses family. In 2002 another SARS coronavirus (SARS-CoV) first infected humans in the Guangdong province of southern China – By 2003 (when it was formerly identified) the epidemic had affected 26 countries and resulted in more than 8,000 cases.^1,2^ Another coronavirus disease known as the Middle East respiratory syndrome (MERS) was first identified in 2012 and caused 858 deaths across 27 countries.^3^ Although COVID-19 is known to have a lower mortality rate than MERS, its quick propagation makes it very dangerous. Some estimates suggest that half of the world's population will be infected and around 100 million will die.^2^

International outbreaks and pandemics of infectious diseases have been present throughout history. In the mid-13th century, the Black Death killed more than 50 million in Europe and the Mediterranean region and 20 million people in India. In 1918-1979 Spanish influenza killed between 50-100 million people.^4,5^ Measles killed 630,000 people in 1990, and Cholera has killed numerous people in seven known historical large outbreaks and continues to have a devastating impact in sporadic cases around the world.^6^

By the end of May 2020, COVID-19 had already spread across the World infecting 5.2 million people and had claimed 337,687 lives. At the time of writing, the United States had the highest number of cases (1,568,448 cases) and the largest death toll of 94,011.^7^ Alarmingly, high case numbers were also reported in the Russian Federation with 344,481 cases at the time of writing. In Europe, the United Kingdom had registered the highest death toll (36,675).^7^ In addition to the mortality and case numbers, the impact of the COVID-19 pandemic has manifested in significant psycho-social and economic burdens, especially in the most affected countries. Such impacts have been caused by restrictions of movement and social gatherings.^8^

China had the earliest experiences of living within this pandemic disease, and important lessons can be learned from their experiences. Currently, China's preventive measures of COVID-19 like wearing masks, hand wash with soap/sanitisers, social distancing, and social gathering avoidance, and isolation, confinement provided promising results.^9^

Africa ranked the last continent and at the time of writing had significantly fewer cases (77,295) and fewer deaths (2,073) where Americas ranked first with 2.3 million cases and 138,116 deaths. Within Africa, South Africa ranked first with 2,343 cases and followed by Egypt with 16,513 cases but with elevated COVID-19 mortality in Africa (735 deaths).^7^ The total cases within the East African Community countries was 2,943 with 78 deaths. At the time of writing, Kenya had registered the most COVID-19 cases (1,192) and deaths (50) compared to other countries of the East African community.^7^

### Case Fatality Rate and ICU Admissions

In February of 2020, the COVID-19 case fatality rate (CFR) in China was 2.3%. In March of the same year, the CFR in Italy was 7.2% mainly due to the high aged population. There was no death recorded in those under 9 years of age, however, the older aged group mortality increased where the group of 70-79 years case fatalities was 12.5% in Italy and 8.0% in China. The group of 80 years and above was 20.2% in Italy and 14.8% in China.^3,10^ The CFR increased to 49.0% (1023 of 2087) in critical cases. It was also elevated for patients with comorbidities among other cardiovascular diseases with CFR of 10.5%, diabetes with CFR of 7.3%; chronic respiratory diseases with CFR of 6.3%, high blood pressure with CFR of 6.0%, Cancer with CFR of 5.6%.

In total, 1,716 of health workers were infected by COVID-19 in China where 14.8% were classified in severe or critical cases, and 5 deaths were recorded.^3,11,12^ In Italy, 16% of all patients were critically ill and required to be taken care of in ICU while only 5% was admitted to ICU in China. The increase of critical status patients in Italy immediately suffocated their health care system especially their intensive-care units. This has emphasised the application of population containment strategies such as lockdown to slow down COVID-19 spreading.^13^

In this viewpoint, we considered the application of COVID-19 prevention, mitigation, and control in low-income countries, especially in Africa. The aim is to assist country response teams in their choice of which evidence-based strategies they could adopt to proactively prevent the exponential growth of the COVID-19 pandemic.

### The Journey from Preparedness to Recovery

This may have speeded the recovery as per the International Health Regulation (IHR) 2005 for minimising multi-sectoral pandemic negative effects.^14,15^ The adaptation of the World Health Organization road map to professional and surge logistic requirement capacity would be oriented to a planned recovery.^16^ Succeeding over the COVID-19 outbreak requires planning, organization, implementation, evidence-based monitoring, and evaluation. The World Health Organization created 7 key points to follow as the next step after the preparedness and response plan. The seven key points of the recovery road map are to (1) appoint lead planners, (2) engage stakeholders, (3) map gaps, (4) address gaps, and (5) engage donors, (6) track progress, and (7) review. The adaptation of these key points will be the recovery cornerstone.^14^

### Lesson Learned From China

China's health emergency response was based on four grades defined according to the incident gravity.^17^ The evaluation of country preparedness and improvement was extremely important to ensure the readiness and outbreak response effectiveness.^18^ The timing of the COVID-19 emergence was challenging, the cases had already steadily increased in China and the government was forced to make difficult decisions around the time of the annual Lunar New Year. During this time more than 1 million people travel to visit their families. The celebration leads to large gatherings, crowded buses, planesand significant movement across the country and globally.^3^ The action taken in China built on lessons learned from the 2002-3 SARS outbreaks, where there was no emergency planning and no mechanism. However, after 2003, China established a plan that was later called: One plan, three strategies which consisted of four-level from top to community leadership.^18^ China focused on traditional public health outbreak response tactics including: isolation; quarantine; social distancing; community containment; local government food provision for effective quarantine.^3^

### The following lessons can be learned from these early responses in China

The Chinese early responses to coronavirus were divided into three stages based on the disease outbreak, the required response, the situation of risk and ecological conditions. The situation of an outbreak emergency was also adapted to the level of emergency response mechanism in respect of assessed emergency grade.^14^ Beijing showed that the emergency response must be based on four-party responsibilities (territorial, departmental, employer and individual responsibilities) which required us to consider the role of the employers and companies (Table1).^19^

**TABLE 1.** The 8 Public Health Prevention Pillars

Pillars	Country Crisis Team	Companies Crisis Team
**Coordination, plan and monitoring**	Initiate multi-sectoral and multi-partner coordination mechanism; Establish public health emergency organs for incident management; Strategic preparedness and response plan along with Country operational preparedness and response plan, Monitoring and evaluation plan (M&E), tracking effectiveness, review, and lessons learned.	Appoint a company crisis team lead; Identify and designate interdepartmental agents to participate in Crisis team; Create Company COVID-19 preparedness and response plan, tracking effectiveness, regular situation report, and work patterns change policies within the company with alignment with the country situation.
**Risk Communication and community engagement**	Initiate multi-sectoral and multi-partner coordination mechanism; Establish public health emergency organs for incident management; Strategic preparedness and response plan along with Country operational preparedness and response plan, Monitoring and evaluation plan (M&E), tracking effectiveness, review, and lessons learned	Company based communication plan, regular communication of policies, memos and all outbreak related information from the crisis team, Administrative work change policies; Prepare and monitor internal information based on rumors or misinformation on the crisis and give real evidence based information to follow guidance and to ensure workplace community engagement; Ensure changes are done and document lessons learned.
**Surveillance, Rapid response and case investigation**	Case definition, Rapid and active case detection and Findings of the imported case and local transmission; Case-based reporting within 24hours, transmission intensity, contacts traceability, disease trend, case fatality ratio; Prepare trained response team for rapid case investigation and contact traceability, test existing system, and document lessons learned..	Prepare manual and electronic reporting forms; Inform the national surveillance system through existing reporting by the occupation clinics if you have the case; Call the national rapid team investigation if there is a case to investigate.
**Point of entry**	Initiate point of entry Public health emergency plan, temperature monitoring and traveling history and associated signs and symptoms, dissemination of current information, Risk assessment screening chart, Standard operating Procedures to manage diseased passengers; Prepare equipped temporary isolation facilities and safe transport to a designated area for COVID-19; Communicate information about travelers, monitor effectiveness, and adjust accordingly.	The point of entry is one of the crucial areas to take care of in the organization; to understand the mechanism of disease entry and who has brought it, and help to know the traceability of his or her contacts (before entry screening chart; Check and monitor temperature and flu-like symptoms would be with capital importance to orient the person to a designed occupational clinic for further investigation); Prepare according to triage guidance with regular review and adjust accordingly.
**National Laboratories**	Guidance of specimen collection and test procedure, surge plan of materials in the needed test, liaise with the international laboratory, trained staff, safety, accessibility with free emergency numbers; Link data with epidemiological analysis and reporting, develop quality assurance, and monitor effectiveness and document lessons learned.	Training of laboratory staff for nasal and oral swab collection and liaise with the national laboratory for the test or if not done on-site liaise with crisis team and national laboratory; Support the national laboratory to get the tests.
**Infection prevention and Control**	Assess Infection prevention and control (IPC) capacity in the healthcare system and in the community: Health workers safety, correct PPE with respect of donning and doffing, Respect aseptic procedure of medical material, surface and objects disinfection, general hygiene monitoring and hand hygiene performance, Gloves usage respect rules of obligatory isolation and terminal hygiene and disinfection; Trained teams to educate people through existing for behavior change such as avoid gathering, observe the social distance, staying home through community networks, local authorities, and monitor effectiveness.	Occupational clinic staff and Medical infrastructure safety and environmental and industrial hygiene respect, surface disinfection, door handles, tables, phones; and other touchable electronic devices using 75% Alcohol based disinfectant or chlorine with concentration caution for avoiding industrial metals oxidation; Educate people for Changing the behavior of handshaking, touching faces or colleagues, community gathering. Ensure the social and physical distancing at least one meter if approaching others; Track the employee's safety against the viral infection.
**Case management**	Record and Follow WHO and CDC directive for Suspect cases, contacts, confirmed cases, health workers with signs and symptoms with a history of treating confirmed cases. Self-isolation to controlled isolation with the designated team; Accurate and timely identification of clinical features in severe risks and appropriate early interventions; Designated ambulance, comprehensive medical, nutritional, and psychological approaches.	Build temporal isolation for the suspect while waiting for the application of governmental hospital directives; Follow governmental emergency directives and call emergency numbers.
**Operational and logistics**	Health professional workforce required existing and new infrastructures such as dedicated hospitals for COVID-19 Outbreak, ICU beds, and functional materials, Medical supplies, test requirement, patients and contacts tracking technologies, water, electricity, another source of energy and food supplies; Surge material preparation Engage with donors, local, and international NGOs.	Ensure their employee's safety, medical requirement and capacity to respect home confinement within the enforced period, help to get food supplies and another health requirement; Surge materials for protection and for essential business continuity; Liaise with local governmental authorities for support and finding out solutions for certain difficulties

#### First stage: Before January 19, 2020: Initial partial control approach.

This phase was dedicated to the investigation, identification of a causative virus, notification, planning, initiation of public health pillars, and provision of initial technical protocol for diagnostic and control.^20,21,22^

- Outbreak announcement;
- Investigation and response by the National Health Commission (NHC) and China CDC;
- Huanan seafood and the wholesale market closed;
- Regular WHO and relevant countries information, the region and China's Hong Kong, Macao and Taiwan about the pneumonia outbreak;
- Implementation of some of 8 public health pillars and technical protocol issuance for Wuhan and WHO notification by the NHC. Emphasis on the point of entry to prevent the exportation and importation of cases for the Hubei surrounding provinces (Table1);
- Wearing a mask to prevent the disease;
- Multisectoral mechanism initiated;
- Completion of gene sequencing by China CDC and isolation of Novel SARS CoV2;
- Adequate logistic preparation for medical equipment and contained people.

#### Second stage: Jan 20 to February 7, 2020: Intensive approach for outbreak intensity reduction.

Comprehensive adoption of various control measures in accordance with the law.^21^

- Establishment of the novel coronavirus infected pneumonia as a notifiable disease and its inclusion into infectious diseases and quarantine law;
- Ensure market supply and all medical requirements;
- Active treatment of cases, deaths reduction in Wuhan;
- Publication of the third version of Coronavirus diagnostic and treatment guideline western and traditional medicine;
- Designation of health facilities for the COVID-19 treatments;
- Contracting five companies for active cases and cluster finding and contact tracing;
- Daily communication on COVID-19 situation by NHC;
- Multisectoral mechanism enhanced by state counsel (social mobilisation, community communication and involvement, NGOs and international community support);
- Implementation of lockdown in Wuhan (traffic restriction and blockade of social-ecological structures such as markets, gatherings, trains, buses);
- Informing WHO and WHO declaration of SARS CoV2 as a public health emergency of international concern;
- Construction of two new Huoshenshan hospitals in Wuhan within one week;
- Development and opening of 16 Fangcang shelter hospitals for treating mild to moderated COVID-19 patients to minimize home transmission took place in home isolation. These hospitals had 3 characteristics (rapid construction, massive scale, and low cost) and were assigned 5 functions(isolation, triage, basic medical care, frequent monitoring, and rapid referral, essential living and social engagement);^22^
- Enhancement of admission and isolated cases treatment in Hubei.
- Environment disinfection;
- Extension of spring festival holiday for social distancing;
- Improvement of the treatment protocol and infection prevention and control(IPC);
- Maintaining the stable supply of commodities;
- Social distancing and hands washing with water and soap/ Use of hand sanitisers;
- Disinfection of surfaces and items such as keys and phones, computer, door handles.

#### Third stage: After February 8, 2020: Rehabilitation

Orderly resumption of production in enterprises^21^

- Maintain a focus on cases treatment and prevent transmission;
- Risk assessment based focus for efforts intensification in regard to the risk of transmission;
- Establishment of the balance between infection prevention measures and socio-economic and sustainable development;
- Health insurance financial compensation;
- Technology-based contact tracing;
- Support of all provinces to Wuhan and other areas in Hubei province;
- Work resumed in phases and pre-school preparation;
- Re-opening of business with continuous social distancing, mask-wearing, hand washing and sanitising with hydro alcohol solution, sneezing and cough etiquette.

Although lockdown was understood to be difficult to be implemented in comparison to all of the other strategies, China managed to successfully apply it.^23^ Lockdown was practically possible and yielded important results in Wuhan by blocking all the key nodes (cities, town, villages, airway lines, railways). Although Kaiser Permanente in Northern California proposed six measures to curb COVID-19, the USA didn't manage to overcome COVID-19.^24^ China, USA, and other resource rich countries have a strong economy, classified to level 5 emergency readiness, high technology and infrastructure, strong health system, but only China experience to 2003 SARS CoV helped the country to contain COVID-19.^16^

Due to the uncertainty of COVID-19 treatment and owing to its long incubation period and the fact that many cases can be asymptomatic; people have been requested to stay home for 14 days of quarantine. This approach has provided indirect protection for uninfected populations by breaking the chain of disease progression; which had a high basic reproduction number (R0) that estimated to be 1.95 by WHO and 2.5 in China. In addition, heard immunity was impossible to realise due to lack of vaccine, and the prohibition exposure to serious fatal disease.^25^

### The possibility of application of lessons learnt from China

Many of the prevention measures performed in China have been successfully applied globally and in Africa as well. The preventive measures such as social distancing, mask-wearing, hand washing and sanitising and handshaking avoidance. The active case, cluster finding and management, contact tracing, point of entry restriction and temperature checking was also applied.^26^ According to CDC Africa, COVID-19 policy improvement and continuous communication by relevant authorities were also helpful to fight rumors. Due to the fact that many low and middle-income countries, especially in Africa have fragile economies, building new COVID-19 hospitals and improving emergency preparedness to level 5 appear difficult. However, the selection of existing structures that could be converted to temporary hospitals could be helpful. This can help to create monitored isolation, limiting community transmission, and elimination of hospital overcrowding.^22^

Drawing from these lessons, there have been great deliberations as to the possibility of population containment in Africa. In many parts of Africa, people live in rural areas where there is less congestion. However, many towns and cities are overcrowded, particularly in urban area.^8^ Hence, in addition to the challenges of fragile economies, the low level of emergency readiness and ability to apply lock down scenarios could be inhibited by lack of infrastructure. According to the Economic Commission for Africa, 42 over 54 countries in Africa have applied full or partial lockdown. They estimated that Africa may lose 2.5% of annual GDP, equivalent to around $65.9 billion for only a one-month full lockdown across the whole continent.^27^ In addition, psychosocial issues, poverty, and hunger, missing routine vaccination may jeopardise the population health if an unplanned full and prolonged lockdown is implemented in African countries.^28^ The resilience features that African countries have today are a young population, hypothesised unfavourable climate to the virus, viral diseases familiarities, and uncongested rural life.^8^ However, international economic support is important to prevent further disasters that may arise as post-COVID-19 lockdown negative effect.^29^

## RECOMMENDATIONS

- Country plan and creation of different level joint teams: Involvement of Country Ministry of Health and World Health Organization; public health professionals, governmental and non-governmental organisations, Military, police, business companies, security organisations and economist;^18,30^
- Protection of frontline health professionals including Doctors, Clinical Officers, Nurses, Midwives and other allied health professionals to avoid panic and health system collapse;
- Isolation preparation for obligatory confirmed cases and non-confirmed cases in follow up;
- Prepare and counts all ICU beds available with functional materials to be early informed on the support capacity already available;
- Raise the red flag for international support;^21,20^
- Creation of government policies through the evolution of Covid-19 and decentralised safety enforcement;
- Strategic and operation Health promotion program and continuous research sharing;
- Establishment of solidarity fund for Social support to help high-risk zone (this was generated by NGOs and from unaffected provinces in China);^21^
- Ensure water availability, electricity and food distribution to people;
- Remote medical management for identification of cases with artificial intelligence by temperature measurement, radiologic examination interpretation.^31^
- Publication of COVID-19 crisis emergency number for anyone who has similar signs of the disease;
- Prepare areas and existing structures to be converted into cheap temporary hospitals like (stadium, gymnasium etc.) for healthy people. This can also eliminate hospital overcrowding as it did in fangcang;^22^
- Lockdown of all international and national points of entry. Except internal based borders pass for Social workers, Food delivery. Healthcare professionals, Emergency team, and patients;
- Confinement (staying at home) is the current strategy to halt the COVID-19. Hence plan it, apply it, follow it, and review it until recovery within the respect of human rights.

## CONCLUSION

Coronavirus disease-19 is currently exasperating the world by increasing cases and subsequent deaths. It has blocked the social-ecological networks (territories, towns, gathering places such market and churches, trains and planes travels) with a broad array of negative impacts (physical, psychological and economic impacts). Therefore, we encourage countries, companies and all organisation entities to apply these Chinese lessons learned for the benefit of the population at large.

We again suggest, for future preparedness, to spot and design places where to install temporary hospitals and isolation structures at the least cost. Establish a team to predict all possible outbreak in ten years and suggest early preparedness and response, and put in place measures of frontline early protection.
